# Effectiveness of Sun Protection Interventions Delivered to Adolescents in a Secondary School Setting: A Systematic Review

**DOI:** 10.1155/2021/6625761

**Published:** 2021-03-04

**Authors:** Bronwen M. McNoe, Kate C. Morgaine, Anthony I. Reeder

**Affiliations:** ^1^Social & Behavioural Research Unit, Department of Preventive & Social Medicine, Dunedin, New Zealand; ^2^Department of Preventive & Social Medicine, Dunedin School of Medicine, University of Otago, Dunedin School of Medicine, University of Otago, Dunedin, New Zealand

## Abstract

**Aim:**

The aim of this systematic review is to summarise the evidence of the effectiveness of interventions targeted to adolescents (13 to 18 years inclusive) and delivered in a secondary school setting with the purpose of improving sun protection behaviour, reducing ultraviolet radiation (UVR) exposure, and/or improving physiological outcomes related to UVR exposure (such as erythema or naevi development).

**Methods:**

Peer-reviewed journal articles were identified from seven database searches (Cochrane, Embase, CINAHL, Scopus, Medline, PsycInfo, and Web of Science) to January 2020, forward citation searches of relevant articles, and monitoring of WHO INTERSUN UVR list server for recent publications. Relevant articles were collected and critically analysed using the Effective Public Health Practice framework. Two reviewers independently reviewed, and when deemed eligible, extracted data and performed quality appraisals for each study.

**Results:**

Thirteen studies met the criteria for inclusion in the review. There were no studies that met a “strong” quality rating, five received a “moderate” quality rating, and eight studies a “weak” quality rating. Three of those with a moderate rating found evidence for effectiveness. The most promising interventions overall (including the pilot/uncontrolled studies) were those that moved beyond a pure health education approach and used innovative approaches such as the provision of shade, or use of technology (e.g., appearance-based apps or real-time ultraviolet index (UVI) monitors).

**Conclusions:**

There is a lack of high-quality published studies investigating the interventions delivered in a secondary school setting to protect students from UVR. The evidence could be strengthened if researchers used consistent, standardised outcome measures for sun protection exposure and behaviour. Other factors limiting the strength of evidence were short follow-up times (largely less than 6 months) and/or nonrobust study design.

## 1. Introduction

Skin cancer is an important public health issue with an estimated 2 to 3 million keratinocytic cancers and 132,000 melanoma skin cancers diagnosed annually worldwide [1]. The population burden of skin cancer mortality and morbidity places a high and increasing cost on treatment services [2, 3]. Yet the risk of developing skin cancer later in life can be diminished by reducing exposure to ultraviolet radiation (UVR) [4].

Although exposure to UVR throughout life is important for skin cancer risk, it may be particularly crucial during childhood and adolescence [5,6]. These age groups spend a considerable amount of their time attending school and the school day encompasses peak UVR time, which is a particularly important issue during the months when it is seasonally at its highest. They can spend at least part of that time outdoors and can consequently receive a substantial proportion of their total UVR exposure while at school [7, 8].

The recommendation of the US Community Services Task Force, which aggregates scientific evidence for use in community-based programmes and policies to improve health, is that skin cancer interventions in primary and middle schools are effective at improving sun protection practices, reducing students' UVR exposure, and subsequently reducing their skin cancer risk [9]. They report that there is insufficient evidence for the efficacy of interventions in high school and college settings, which were assessed as one entity as both settings are similarly structured in the US [10]. For other parts of the world, including New Zealand (NZ), the secondary school setting is very structured, but the university setting is much less so. Accordingly, that review is not optimal for such contexts.

The aim of this systematic review is to summarise the evidence about the effectiveness of interventions targeted to adolescents (aged 13 to 18 years inclusive) and delivered in a secondary school setting with the purpose of improving sun protection behaviour, reducing UVR exposure and/or improving physiological outcomes related to UVR exposure (such as erythema or naevi development).

## 2. Methods

The Effective Public Health Practice Project (EPHPP) process for systematic review was followed [11]. This method allows for the assessment of both randomised controlled trials (RCTs) but also nonrandomised studies specifically in public health and health promotion.

The lead author (together with one of the coauthors, Associate Professor Reeder or Dr. Morgaine) was involved in the literature search, study selection process, data extraction, and quality assessment. At each point of the review process, two reviewers (BM and either AR or KM) independently made the assessment. Any differences were resolved after discussion with the third reviewer (AR or KM) and consensus was reached.

### 2.1. Question Formulation

Adhering to the EPHPP guidelines for question formulation, the research question developed was as follows:

Is there evidence for the effectiveness of interventions for adolescents in secondary school settings which aim to

- Improve sun protection behaviour?
- Achieve a reduction in UVR exposure?
- Improve physiological outcomes related to UVR exposure (such as erythema or naevi)?

### 2.2. Searching Literature

**Figure 1:**
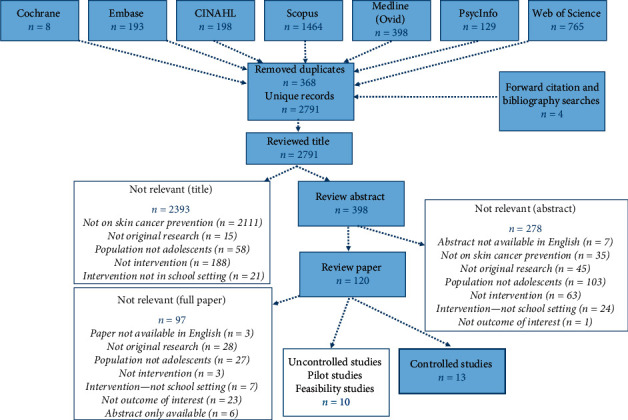
Search strategy—interventions delivered in secondary school for adolescents.

A comprehensive systematic search of the published literature on interventions delivered in a secondary school setting for the prevention of skin cancer in adolescents was conducted using relevant index search terms which encompassed age group, school setting, interventions, sun protection behaviour, and outcomes of interest (Supplementary File 1). The databases searched were CINAHL, Cochrane, Embase, Medline, PsycInfo, Scopus, and Web of Science (Figure 1). All epidemiological studies published up to 30 January 2020 were included (the search was not limited to a start date so that all literature was documented). In addition, for articles identified for inclusion, reference lists were examined as well as a forward citation search conducted. Two studies published after the cut-off date of 30th January were identified in this way. The INTERSUN UVR list server, an important communication tool facilitated by the WHO and frequented by many international experts in the field, was also actively monitored to identify recently published literature. Unpublished material was not considered as it is not always easily accessible and is not generally peer reviewed. All records identified from the search process were exported to an EndNote^TM^ referencing database. Duplicates were identified and removed, and the remaining records were screened first by title and then by abstract, independently by two reviewers. A conservative approach was used to ensure that no potential studies were missed. All articles judged potentially relevant and meeting the eligibility criteria were reviewed in full text.

### 2.3. Study Selection

#### 2.3.1. Population of Interest

The population of interest was adolescents (13–18 years inclusive). Interventions not exclusively targeted to adolescents (for example, target group, 10 to 15 years) were only included if results for adolescents were presented separately.

#### 2.3.2. Interventions

Each intervention needed to be either delivered in a secondary school setting exclusively, or if in a combined primary and secondary school setting, the results from the secondary school needed to be separately reported. Interventions delivered outside the school setting (such as online) were excluded. Both single-component and multicomponent interventions were potentially eligible.

#### 2.3.3. Outcomes

Interventions that aimed to either improve sun protection behaviour and/or reduce UVR exposure or skin damage were included in the review. Only papers with outcomes of “actual behaviour,” UVR exposure, or physiological outcomes were included. These were sun protection behaviour (use of sun protective hats, clothing, sunglasses, sunscreen, or shade), reduced UVR exposure (staying indoors during peak UVR, or reduction in objectively measured UVR, self-reported intentional tanning or sunbed use), and physiological outcomes (erythema, naevi development, skin damage, or skin cancer development). Articles where the outcome measures were exclusively knowledge, attitudes, or intended behaviours were excluded as these did not necessarily result in behaviour change [12], reduction in UVR exposure, and subsequent decreased risk of skin cancer.

## 3. Study Design

Randomised and nonrandomised controlled studies, and those implemented before-after study designs, were included in the quality assessment review. Those papers reporting on uncontrolled or pilot/feasibility studies were excluded from quality review and evidence tables as these are considered weak design or underpowered for detecting statistical differences between groups. However, we considered it important to still report these studies as they can potentially provide interesting and innovative ideas for interventions that could be tested for effectiveness using a more robust study design. To be eligible, articles needed to be available in the English language. Non-English articles were excluded at the stage where an English translation was not available (i.e., at title, abstract, or full paper stage). This enabled interventions delivered in languages other than English to be documented. Articles not providing original data (editorials, letters, and reviews) were excluded.

### 3.1. Determination of Study Quality

The EPHPP tool was used to assess six aspects of intervention quality (Table 1): selection bias, study design, confounding, blinding, data collection, and withdrawal/dropouts. Each dimension is ranked on a three-point Likert-type scale (“weak”, “moderate”, and “strong”) according to EPHPP guidelines [11]. A total rating based on the individual ratings of the individual components was calculated. Studies with a minimum of four “strong” and no “weak” ratings were classified as “strong.” Those with four “strong” and one “weak” rated as “moderate”, and those with two or more “weak” rated as “weak” [11].

### 3.2. Data Extraction and Synthesis

A standardised protocol was used to extract the data from eligible articles. This included first author, data collection period, study design, geographical area, target population, sampling frame, sample population (including demographic characteristics), theoretical framework, intervention (format, content, duration, and delivery), control group, follow-up period, and results. Two reviewers (BM and KM) completed the data extraction and any discrepancies were reviewed and resolved by discussion involving all three reviewers.

### 3.3. Data Synthesis

The information from the data extraction was synthesised narratively, but not quantitatively, because of the large variability in the outcome measures.

## 4. Results

Figure 1 summarises the study selection process. A total of 3,159 records were retrieved when combining the results from each of the seven database searches along with the forward citation and bibliographic searches. After removal of the duplicates (*n* = 368), 2,791 unique records were identified. Of these, 2,393 were removed based on the title and 278 on the abstract, leaving 120 papers for full review. After fully reading the papers, 97 records were identified as not meeting the eligibility criteria. In a further 10 articles, the study design was either a pilot study or there was no control group, so although these are described, they did not undergo quality review. The remaining 13 papers were assessed for data quality.

### 4.1. Study Quality

Of the 13 studies, five were classified as “moderate” [13–17] and eight as “weak” [12, 18–24] according to the EPHPP quality criteria. There were no studies rated as “strong” as they all had at least one “weak” component rating. *Selection bias* was particularly prevalent in getting “weak” ratings. Overall, eight of the 13 studies were rated as “weak” largely because either the study design did not include randomisation of schools and/or the rate of participation of schools and/or students was low (classified as under 60% of those invited). The other notable area which negatively impacted on all the study ratings was the *blinding* of assessors and participants. All but one of the studies included self-reported outcome measures, and so the rating component of observer bias was not relevant. Therefore, the rating for *blinding* was solely dependent on whether the participants were aware of the research question. None of the studies explicitly stated whether or not this was the case, and so all these studies achieved a rating of “moderate” as per the EPHPP protocol. All but one of the studies used questionnaires to assess outcome measures. Where *validity* or *reliability* for the questionnaires was not provided, the study received a “weak” rating with respect to data collection. In all cases, the *study design* was well described, and for most either important potential *confounding factors* were shown to be balanced at baseline between groups or were controlled for in data analysis. Although not specifically part of the quality review, some studies reported *p* values between the intervention and control group, but did not go on to provide the time by condition effect. Most of the studies also provided a good description of *withdrawals/dropouts*, with the use of flowcharts adding clarity in many.

Characteristics of the studies reviewed are presented in Table 2 with a more detailed summary in Supplementary File 2. The characteristics of uncontrolled and pilot studies, not considered in the quality review, are presented in Table 3 with more details provided in Supplementary File 3.

### 4.2. Intervention Characteristics

The geographical regions where the intervention was delivered were globally diverse; three studies each in Australia [12, 15, 23] and Iran [14, 16, 17], two in the United States [22, 24], and one each in Brazil [14], China [20], Denmark [18], Spain [25], and Turkey [21]. Of the 13 studies, seven reported that they were based on a theoretical framework which included Protection Motivation Theory [13, 17], the PRECEDE model [16], the Health Belief Model [22], the Theory of Planned Behaviour [23], the Extended Parallel Process Model [24], or some combination of behavioural frameworks [12]. All but one [15] of the interventions included didactic educational material on sun protection and the risk of sun exposure. Of the 10 studies for which information was provided on who delivered the intervention, half were delivered by the research team [13, 16, 23, 24] or a medical student [14], two were delivered in the classroom using online [19] or video material [22], two by the classroom teacher [12, 18], and one was an environmental intervention [15]. In addition to lecture material, five interventions had an interactive component such as games, role playing, or appearance-focused apps or photographs [14, 19, 23, 24], [12]. Some also specified that they included take-home materials such as brochures or magazines [21]. One intervention was entirely environmental in that it was the provision of shade sails in the playground [15]. Of the12 studies where duration of the intervention was provided, five were single sessions of less than one-hour duration [14, 19, 21, 22, 24]. The remaining interventions were longer with six reporting multiple sessions, usually over a block of time [12, 13, 18, 23] with one exception where two lessons were delivered each year for three years [20]. The intervention where shade was provided was long-term [15].

### 4.3. Study Characteristics

The majority (77%) of the studies were RCT [12–16, 18–20, 22, 23] with the other three being either a nonrandomised trial [21, 24] or a controlled pre/postintervention [17]. Length of follow-up was short, with 10 studies being six months or less (ranging from four weeks to six months) [13–19, 21–24]. Of the two studies with longer follow-up times, one was for two years [20] and the other three years [20]. The one study which comprised an environmental intervention of the provision of shade sails had continuous follow-up for 14 weeks [15].

### 4.4. Participant Characteristics

Most of the interventions (86%) were delivered across all years of secondary school for students from age 13 to 18 years [13–21, 23, 24]. Two studies delivered the intervention to a single year group [12, 22]. For studies where information on the sex of participants was reported (*n* = 11), two were at single-sex schools (one each for male [16] and female [13] students), the proportion of females was higher in two studies (61–76%) [22, 23] and lower in one study (10%) [21]. The participants in the remaining studies were approximately half female and half male [14, 17–20, 24]. Skin colour or ethnicity was not well reported with only five studies [14, 19, 22–24] collecting this information and responses varying greatly by country where the intervention was delivered.

### 4.5. Quality Rating

Four of the five studies with a “moderate” rating contained an educational lecture component with three interactive activities. In addition to a single lecture, Brinkler et al. provided students in Brazil with selfies altered by an application to show the effects of UVR exposure on their faces 5–25 years in the future [14]. They reported an improvement in the self-reported use of daily sunscreen but no reduction in intentional tanning over the six-month follow-up. The three interventions delivered in Iran [13, 16, 17] were either multiple educational sessions or an educational session in combination with a take-home pamphlet [17]. All used varying sun protection behaviour scores, but only Jeihooni reported a significant improvement [16]. The follow-up period was short ranging from 2 to 4 months.

The remaining study with a “moderate” rating was an Australian intervention that was completely environmental with no educational component [15]. Direct observation was used to assess the use of shade sails by students at the school and compare this to an alternate site where there was no shade sail. Although the numbers were small, there was a significant improvement in shade use over the 14-week follow-up period.

### 4.6. Pilot or Uncontrolled Studies

Almost all (nine of the 10 studies) of the studies were similar to those seen in the previous section in that they were educational lectures with or without interactive activities. One exception was an Australian study by Pettigrew et al. [32], which combined an educational presentation in school assembly with environmental education, which was a real-time UVI monitor placed in the playground.

## 5. Discussion

UVR exposure during adolescence is an important risk factor for the development of skin cancer later in life [36, 37]. Well-designed interventions informed by “best evidence” could potentially reduce this exposure. The obvious setting for the delivery of interventions among adolescents is where they commonly assemble, that is, in the school setting. This review adds to other systematic reviews on interventions targeting a reduction of UVR in various populations and settings [38–40]. To the best of our knowledge, this is the first systematic review of this nature specifically focusing on interventions that target adolescents in a secondary school setting. Adolescents as a group face unique challenges that are different to other age groups and secondary schools are unique contexts for potentially addressing these challenges.

It is preferable for interventions to be designed using theoretical foundations as this takes into account the mediating factors which are influential in achieving behavioural change [41]. Encouragingly, over half of the interventions reviewed were designed using a behavioural theoretical framework such as Protection Motivation Theory which describes how, when confronted with a perceived health threat, an individual is motivated to react [42].

Thirteen studies were included in the review; however, over half of the reported studies were found to have methodological limitations that affected their overall quality rating. None of the studies received a “strong” quality rating, and only five had a “moderate” quality rating according to the EPHPP assessment criteria. The outcome measures were similar in each study in that they covered the use of sun protection items (sunscreen, hats, clothing, shade, and sunglasses), UVR exposure (intentional tanning and sunbed use), or physiological outcomes (sunburn). The way in which outcome measures were collected makes comparisons between studies problematic beyond describing whether significant results were seen. For example, in some studies, a summative score (based on between four and 10 individual behaviours) of sun protection behaviours was provided, whereas in others individual sun protection behaviours were reported. It is difficult to interpret a study where a significant effect was observed for one sun protective behaviour but not others. As noted in other reviews, the follow-up time assessing the impact of interventions was relatively short, with the majority being less than six months, so the sustainability of an intervention cannot be assessed [40].

The interventions were generally well described and remarkably homogenous in content, largely consisting of educational materials delivered in a lecture format in the classroom by a single presenter. Additional components included take-home materials or interactive activities such as those focused on appearance-based activities with apps or photographs. Appearance-based interventions have previously shown promise in the slighter older college age population [43, 44]. A single study used the environmental intervention of the provision of purpose-built shade for sun protection within the school environment.

The types of interventions showing the most promise in improving outcomes were those that moved beyond pure health education. Even if effective, educational delivered interventions are resource heavy in that they require ongoing input from researchers or school staff for every cohort of students. It is important when evaluating interventions to consider whether they could practically be scaled up from a scientific study to a real-world situation. In NZ, despite high rates of skin cancer, a relatively high UVI, a large proportion of the population with light skin, poor sun protection practices, and an outdoor lifestyle, sun protection is not regularly taught within the secondary school curriculum [45]. This is likely, at least, in part due to the number of well-being related topics that need to be covered in secondary school, some with more immediate consequences for health than sun exposure. Innovatively, the Australian Cancer Council, which has led the world in skin cancer control, has developed two interventions which are environmentally based and thus would not need ongoing resources, although both did include a small educational component for students [15, 32]. These were either the provision of shade sails or a real-time UVI monitor in the playground. Australian children have benefited from SunSmart messages from the start of school and so may be better educated on the dangers of UVR and interpreting the UVI than children of other nations. However, both of these environmental interventions need to be tested further, both in Australia and elsewhere, to assess their efficacy, generalisability, and sustainability. In addition to the articles reviewed, one study reported on a feasibility study of an educational intervention combined with text message reminders to students [46]. This study did not meet the inclusion criteria because being a feasibility study, outcome measures were not reported. Innovatively, this study tested objective outcome measures of melanin and erythema using a Mexameter [46].

### 5.1. Strengths and Limitations

All published studies reporting interventions designed to reduce UVR exposure among adolescents have been reported, regardless of the study quality or design. These may provide novel ideas for interventions that could either be scaled up or adapted and evaluated using more robust study methods. There are several limitations associated with this systematic review. First, only published studies were considered, thereby introducing the potential for publication bias. Second, the way that outcome measures were reported in the studies was diverse, making comparisons difficult. Furthermore, the majority of the studies were based on participants' self-reported outcome measures that may be subject to both social desirability and recall bias and frequently without validity and reliability measures. There is starting to be an increase in studies based on observational outcome measures, which is a positive trend. Third, although the geographic locations were varied, it was not possible to determine the effect these interventions might have in locations with different climates or populations of varying skin colours. Fourth, it was not possible to disaggregate the different components of the intervention nor fifth, the intensity of the language used, which is thought to be important in the efficacy of messaging [47].

## 6. Conclusion

There is a lack of high-quality published studies investigating interventions delivered in a secondary school setting to protect students from UVR. Most sun protection interventions targeting adolescents are health-based, emphasising the association between UVR exposure and skin cancer. These have limited success in changing behaviour. Two promising interventions were identified as potentially relevant for adolescents. Both moved beyond presenting didactic education in the classroom. The first was an appearance-based intervention using facial ageing technology in the classroom. The second was an environmental intervention with the provision of a real-time UVI display meter in the school playground.

The evidence generally could be strengthened if researchers used a consistent, standardised outcome measure for sun protection exposure and behaviour, such as that proposed by Glanz et al. in 2008 [48]. Other factors limiting the strength of evidence were short follow-up times (largely less than 6 months) and/or nonrobust study designs.

## Figures and Tables

**Table 1:** Quality assessment components and ratings for EPHPP instrument.

Components	Strong	Moderate	Weak
Selection bias	Very likely to be representative of the target population and greater than 80% participation rate	Somewhat likely to be representative of the target population and 60–79% participation rate	All other responses or not stated
Design	RCT and CCT	Cohort analytic, case-control, cohort, or an interrupted time series	All other designs or design not stated
Confounders	Controlled for at least 80% of confounders	Controlled for 60–79% of confounders	Confounders not controlled for, or not stated
Blinding	Blinding of outcome assessor and study participants to intervention status and/or research question	Blinding of either outcome assessor or study participants	Outcome assessor and study participants are aware of intervention status and/or research question
Data collection	Tools are valid and reliable	Tools are valid but reliability not described	No evidence of validity or reliability
Withdrawals/dropouts	Follow-up rate of >80% of participants	Follow-up rate of 60–79% of participants	Follow-up rate of <60% of participants or withdrawals and dropouts not described

Reproduced with permission [11].

**Table 2:** Studies identified in the systematic review and reviewed^+^.

First author Data collection period Study design	Global rating (EPHPP)	Target population Sample size Demographic characteristics	Theoretical framework Intervention Control	Follow-up	Result measure Statistical improvement (in at least one intervention and one follow-up time)*∗*
Baghianimoghadam [13] 2009 Cluster RCT	Moderate	*Target population:* High school students—Yazd, Iran *Sample size:* Schools—4 Students—360 *Demographic characteristics:* Age (years): mean 16.04 (0.98) Sex: female—100% Ethnicity/skin colour: not provided	*Theoretical framework:* Protection motivation theory **Intervention—individually directed** *Format:* Lecture, group teaching and performance *Content:* Education (not further described) *Duration*: 3 × 1 hour sessions *Delivery:* Researchers **Control** Wait listed intervention	2 months	**Behaviour score (self-report)** *∗* **Individual sun protection measures (self-report)** Sunscreen*∗*, sunglasses*∗*, gloves*∗*, hat*∗*, clothes*∗*

Brinkler [14] 2018 Cluster RCT	Moderate	*Target population:* High school students—Brazil *Sample size:* School—8 (52 classes) Students—1,573 *Demographic characteristics:* Age (years): mean 15.9 (1.3) Sex: female—51.6%, male—48.4% Skin type: 7.4% I or II, 34.9% III, 50.2% IV, 7.6% V	*Theoretical framework:* None mentioned **Intervention “Sunface” — individually directed** *Format:* Application (app) and education in classroom setting *Content:* Adolescents' selfies were altered by an app to show UVR effects on their future faces (taking into account skin type) and were shown in front of their class, accompanied by information about UVR protection. The app encompasses the effects of UVR on photoaging of the skin in general and the development of skin cancer. *Duration:* 45 minutes *Delivery:* Medical students **Control** No intervention received	6 months	**Individual behaviour (self-report)** Sunscreen*∗*, tanning*∗*

Dobbinson [15] 2004–2006 Cluster RCT	Moderate	*Target population*: Secondary school students—Melbourne, Australia *Sample size:* Schools—51 *Demographic characteristics:* Not collected	*Theoretical framework:* None mentioned **Intervention—Environmental directed** *Format:* Environmental *Content:* Two full sun areas in each school (1 intervention (primary) and 1 alternate site) had building shade sail structures installed (at intervention site only) for students to use during passive activities such as eating lunch (mean cost A$11,500 of shade sail and installation costs varied, maximum $22,000) *Duration:* NA *Delivery:* Environmental **Control** No intervention received	Continuous 14 weeks	**Individual Behaviour (direct observation)** Shade seeking*∗*

Jeihooni [16] 2016–2017 Cluster RCT	Moderate	*Target population*: High school students—Fasa city, Iran *Sample size:* Schools—4 Students—300 *Demographic characteristics:* Age (years): IG: 16.05 (1.76), CG: 16.20 (1.71) Sex: 100% male Ethnicity/skin colour: not provided	*Theoretical framework:* PRECEDE model **Intervention** *Format:* Educational session including group discussion, questions and answers, practical presentation, use of videos, PowerPoint presentation, instruction booklet. *Content:* Education—skin health, skin cancer and risks, sunlight, sun protection. *n.b. Telephone group also organised for students parents* *Duration:* 6 training sessions of 45–50 minutes duration held on weekly basis *Delivery:* Research team **Control** No intervention received	4 months	**Behaviour score (self-report)** *∗*

Rahmatiasl [17] Year not provided Pre/post (control group)	Moderate	*Target population:* 1st-grade high school students—Ahwaz, Iran *Sample size:* Schools—4 Students—215 *Demographic characteristics:* Age (years): 1st grade (13 years) Sex: 47.9%—females, 52.1%—male Ethnicity/skin colour: not provided	*Theoretical framework:* Protection motivation theory **Intervention** *Format:* Lecture and question and answer session. Pamphlet also distributed. *Content:* UVR, UVR and health effects of exposure to UVR, the factors affecting the exposure to UV light, the importance of sun protection in childhood and adolescence, how to protect from the sunlight, benefits of using protective devices against the sunlight and correct ways to use sunscreen. *Duration:* Not specified *Delivery:* Not specified **Control** No intervention received	4 months	**Behaviour score (self-report)** *∗*

Aarestrup [18] 2010–2011 Cluster RCT	Weak	*Target population*: 14–18-year-old students—Denmark *Sample size:* Schools—33 Students—3,635 *Demographic characteristics:* Age (years): 14–8%, 15–42%, 16–47%, 17–3% Sex: female—51% Ethnicity/skin colour: not provided	*Theoretical framework:* None mentioned **Intervention—individually directed** *Format:* E-magazine, short films, advertisements, campaign materials, paintings, social media, poetry, fiction & literature *Content:* Health risks associated with sunbed use as well as appearance damaging effects *Duration:* Mean 5.6 lessons per class *Delivery:* Classroom teacher—teachers guide provided **Control** No intervention received	6 months	**Individual behaviour (self-report)** Sunbed use*∗*

Buendia-Eisman [25] 2009 Cluster RCT	Weak	*Target population:* Students 12–16 years—Andalusia, Spain *Sample size*: Schools—12 Students—1,290 *Demographic characteristics:* Age (years): 12–16, mean 13.75 Sex: 49.8% female, 50.2% male Skin type: 11.5% I or II, 76.8% III or IV, 11.8% V or VI	*Theoretical framework:* None mentioned **Intervention “Healthy sun Habits”—individually directed** *Format:* Online web page *Content:* Webpage structured *∗*The sun—sun & UVR characteristics, dangers of sunburn *∗*sun without danger—emphasised factors associated with sunburn and appropriate sun protection behaviours *∗*Key sun protection messages *∗*Games and website links *Duration:* Pupils used the website for at least 1 hour at school and then had it available for use through the summer. *Delivery:* Entirely on the Internet with teachers only providing technical support for using web page. **Control** No intervention received	3 months	**Physiological measures (self-report)** Sunburn*∗*, sunburn with blisters **Individual behaviour (self-report) -**sunbathing, sun protection measures, sun protection cloudy day*∗* Sun cream

Lai [20] 2012–2014 Cluster RCT	Weak	*Target population:* High school students aged 12–18 years in Beijing, China *Sample size*: Schools—3 Students—638 *Demographic characteristics:* Age (years): mean 14.4 (2.5) Sex: 51%—female, 49%—male Ethnicity/skin colour: not provided	*Theoretical framework:* None specified **Intervention** *IG1* *Format:* Presentations, photograph, and pamphlets *Content:* (i) Education*—*nature and dangers of UVR, sun protection methods, correct use of sunscreen (ii) A photograph was taken of students to assess skin type and students were taught how to protect themselves according to skin type and UV index (iii) Pamphlets contained highlights of presentation *Duration:* 2 × 45 minutes per year (over 3 years) *Delivery:* Received 2X during year 1 and year 2 *IG2* *Content:* Educational pamphlet (as IG1) *Format:* Pamphlet *Duration:* Received 2X during year 1 *Delivery:* NA **Control** No intervention received	2 years	**Physiological outcomes (self-reported)** Sunburn*∗*, suntan*∗* **Individual behaviour (self-report) -**sunscreen*∗*, protective clothes*∗*, hats*∗*, sun umbrella*∗*, sunglasses, avoiding sun exposure*∗*, seeking shade*∗*

Lowe [12] 1993–1995 Pair RCT	Weak	*Target population:* Grade 8 high school students Queensland, Australia *Sample population*: Schools—26 Students—3400 *Demographic characteristics:* Age (years): grade 8 (13 years) Sex: not provided Ethnicity/skin colour: not provided		3 years	**Behaviour score (self-report)∗**

Sumen [21] 2013 Nonrandomised trial (with control group)	Weak	*Target population:* maritime high school students—Antalya, Turkey *Sample population*: Schools—2 Students—567 *Demographic characteristics:* Age (years): 14–2.6%, 15–25.7%, 16–31.7%, 17–25.4%, 18–14.6% Sex: 10.1% female, 89.9% male Ethnicity/skin colour: not provided	*Theoretical framework:* None specified **Intervention** *Format:* Didactic (classroom), brochures (take-home), and posters (environment) *Content:* Training regarding skin cancer, sun protection steps, and harmful effects of the sun followed by “Dear 16-year old me” video which emphasises the importance of sun protection in the adolescent period. Educational material also provided to students at the end of training session. Four weeks following training posters were hung within the school and classrooms as a reminder. *Duration:* 35–45 minutes *Delivery:* Not specified **Control** Wait listed intervention	3 months	**Individual behaviour (self-report)** Sun protection cream∗, sun protection cream – beach∗, sun protection cream – long time outdoors∗, sun protection factor above 20∗, remain in shade∗, stay indoors∗, sunglasses∗, clothing – shoulders, sun protective hats

Tuong [22] 2012 RCT	Weak	*Target population:* 11 grade students—California, USA *Sample population*: School—1 Students—50 *Demographic characteristics:* Age (years): ∗IG: mean age 17.1 (0.88) CG: mean age 17.2 (0.44) Sex: IG: 76% female, 24% male CG: 84% female, 16% male Skin type: IG: 12% White, 88% non-White CG: 4% White, 96% non-White	*Theoretical framework:* Health Belief Model **Intervention** *Format:* Integrated into the classroom health education, viewed video assigned as a group *Content:* Appearance-based video on UV induced premature ageing *Duration:* video 5-minute duration *Delivery:* Assume classroom teacher **Control** Health-based video emphasising UV exposure and skin cancer risk	6 weeks	**Individual behaviour (self-report)** Sunscreen∗, shade, hat, long sleeved shirt,

White [23] Date not provided Cluster RCT	Weak	*Sampling frame:* High school students—Queensland, Australia *Sample population*: Schools —9 Students—382 students (analysed 213) *Demographic characteristics:* Age (years): mean 13.73 Sex: 61.1% female, 38.9% male Skin type: 59% very fair or fair	*Theoretical framework:* Theory of Planned Behaviour **Intervention** *Format:* group-based discussions, role playing, and goal setting. *Content:* Session 1: sun protection related attitudes and beliefs, long/short-term effects of sun exposure and advantages and disadvantages of sun protection Session 2: Foster perceptions of normative beliefs on sun protection. Session 3: Aimed to increased perceptions of self-efficacy over using sun protection measures *Duration:* 1 hour per week for 3 weeks *Delivery:* Facilitated by Cancer Council Queensland staff **Control** Wait-listed intervention	4 week follow-up	**Individual behaviour (self-report)** Sunsafe behaviour weekend∗, Sunsafe behaviour—weekday

Wu [24] 2017 Cluster (school) non randomised trial (with control group)	Weak	*Target population:* High school students grades 9-12—Utah, USA *Sample population*: Schools—11 Students—1,573 students *Demographic characteristics:* Age (years): 9th grade—26.2%, 10th grade 52.3%, 11th grade—12.1%, 12th grade—9.3% Sex: 49.5% female, 50% male, 0.5% other Race: 62.5% non-Hispanic White, 25.8% Hispanic, 2.8% African American, 2.0% American Indian, 3.2% Asian American, 3.7% other	*Theoretical framework:* Extended parallel process model—communicate health risk and prevention information by targeted individual's perceived threat and perceived efficacy. **Intervention** **IG1** *Format:* Health or science class, PowerPoint and interactive activity (3-4 students per group), classroom discussion *Content:* Education (see CG) plus a sunscreen activity which illustrates the UVR blocking properties of sunscreen of differing SPF levels *Duration:* 1 classroom period *Delivery:* Research assistants **IG2** *Format:* Health/science class *Content:* Education (see CG) and receipt of a printed personalised photograph showing current skin damage cause of UVR exposure. Class discussion on how these photos related to UVR dam GE and skin cancer risk. *Duration:* 1 classroom period *Delivery:* Research assistants **IG3** *Format:* Health/science class, PowerPoint and interactive activity (individual) *Content:* Education (see CG) and behavioural change worksheet aimed to improve self-efficacy using sun protection goal setting and planning. Students first selected a behaviour they were willing to commit to implementing in the next month *Duration:* 1 classroom period *Delivery:* Research assistants **Control** *Format:* Health/science class— PowerPoint and interactive activity (individual), classroom discussion *Content:* Skin cancer education—incidence, risk factors, causes, strategies to prevent and screen, common misconceptions, and prevention strategies *Duration:* 1 classroom period *Delivery:* Research Assistants	1 month	**Individual behaviour (self-report)** Sunscreen∗, long-sleeved shirt∗, long pants or skirt∗, wide brimmed hat∗, shade or umbrella∗, avoid peak hours∗, sunglasses, indoor tanning∗, outdoor tanning∗ **Physiological behaviour (self-report)** Sunburn∗

^+^More detailed information and full results are provided in the Supplementary File 2.

**Table 3:** Studies identified in the systematic review but not reviewed (pilot/uncontrolled)^+^.

Author Data collection period Study design	Theoretical framework Intervention Control
Davis [26] 2010 Pre/post (uncontrolled) (pilot study)	*Theoretical framework:* Health Belief Model—specifically cue to action and increase self-efficacy **Intervention—“project students are SunSafe”** *Format:* PowerPoint presentation and three interactive activities in classroom setting *Content:* Education—Epidemiology, skin structure, skin cancer types, UVR, sun protection strategies, tanning consequences. ^∗^Perceived susceptibility—video testimonial of 11-year melanoma survivor. ^∗^Perceived severity of UVR skin damage—images of overexposed individuals are presented (including celebrities). ^∗^Benefits of not engaging in indoor tanning—displaying items that could be bought in exchange for money not spent in tanning booths. Interactive activities—students shown skin through filtered UVR analyser, sunscreen, UVR detecting Frisbee used to visualise protection provided by different fabrics *Duration:* 50–65 minutes (25 minutes of this is PowerPoint) *Delivery:* University students (trained in sun safety and presentation)

Geller [27] 2001 Pre/post (pilot study)	*Theoretical framework:* “Theory driven” exact theory not specified **Intervention “SunSmart America”** *Format:* Education—science (biology) classes *Content:* Cancer prevention and detection curriculum integrated into school biology classes (based on SunSmart Australia curriculum for Victorian schools in Australia)—what is cancer, types of cancer, are you SunSmart? The genetics of skin cancer, sunburn and the UV index, natural selection and skin colour, SunSmart health habits *Duration:* Choose 7 of 12 modules—45–60 minutes in duration *Delivery:* Classroom teacher

Hughes [28] 1990 Postintervention (controlled)	*Theoretical framework:* None specified **Intervention** *Format:* Leaflet. Workbook and video IG1—education + workbook IG2—education + workbook + video IG3—education + workbook + homework to design posters IG4—education + additional discussion later in the week *Content:* education modules in health and physical education classes delivered. Materials: (i) Colour leaflet designed to make covering up look cool. (ii) Workbook contained information on sun, UVR, and skin cancer. (iii) Video—celebrity discusses concepts of sun and skin cancer with class of children. *Duration:* No information *Delivery:* Classroom—research team

Kouzes [29] 2015–2016 Pre/post (uncontrolled) (pilot)	*Theoretical framework:* None specified **Intervention “SunSmart schools pilot program”** *Format:* Curriculum delivered in health class, environmental—provision of sunscreen *Content:* (i) Schools were asked to implement a written sun protection policy. School were given flexibility with which aspects of the program to implement, options included the following: (ii) Age-relevant evidence-based curriculum materials—evidence based, easy to use and teach, and met schools common core requirements (iii) Daily UV index announcements (iv) Guest speaker (v) Provision of sunscreen (vi) Allowing students to wear hats and sunglasses. *Duration:* Not specified *Delivery:* School staff

Loescher [30] December 2016–March 2017 Pre/post (uncontrolled)	*Theoretical framework:* None specified **Intervention “Project Students are SunSafe” (adapted for Hispanic/Latino students)”** *Format:* Classroom lesson *Content:* ^∗^Education—PowerPoint presentation—included information on sunscreen ingredients ^∗^Three activities—interactive sun protective fabric. Sunscreen ingredient or skin analyser activity *Duration:* 40 minutes (3 modules took no longer than 20 minutes to deliver) *Delivery:* Students from school were trained as peer educators to implement the lesson (in groups of 3) to their peers at their school

Milijkovic [31] 2007/2008 (2 periods of data collection) Pre/post (uncontrolled)	*Theoretical framework:* None specified **Intervention “sunbathing—yes or no?”** *Format:* Lecture and workshop *Content:* ^∗^Lecture covered; solar radiation—wavelengths, damage of the ozone layer and its health-related effects, beneficial effects of sunbathing, adverse effects of UV exposure from sun and/or sunbeds, proper behaviour at the exposure, UVI, sunscreens for beach and daily skin care products, sunbeds—legislation, WHO recommendations, artificial skin tanning products, and postexposure skin care and products. ^∗^Workshop—students were given several everyday situations and asked to apply what they learnt from the lecture *Duration:* 90 minutes *Delivery:* Not stated

Pettigrew [32] 2019 Pre/post (pilot study)	*Theoretical framework:* None specified **Intervention—individually directed plus environmental** *Format:* Presentation was in assembly, environmental UVR monitor *Content:* ^∗^Presentation: Purpose of UVI, threshold of 3 for when sun protection is indicated accompanied by graphical content and actionable messages ^∗^Environmental: UVR monitor with attached sign featuring a graduated call to action *Duration:* Presentation 15 minutes long *Delivery:* Not stated likely research team

Ramstack [33] Year not provided Pre/post (uncontrolled)	**Intervention group** *Format:* Didactic (workbooks)—interactive and activity based *Content:* Teacher could choose activities from within each unit: The sun, The skin, The sun friend or foe?, Cancer and skin cancer, Prevention of sun damage *Duration:* 6 units—no details on how frequently delivered or length *Delivery:* Delivered by classroom teacher (training provided)

Swindler [34] Year not provided Pre/post (uncontrolled)	*Theoretical framework:* Not mentioned **Intervention group—individually directed** *Format:* PowerPoint lecture *Content:* Educational—risks of sun exposure and its contribution to premature aging and skin cancers and effective sun protection including sun avoidance during peak hours, sunscreen knowledge, and sun protective effect of clothing. *Duration:* 45 minutes *Delivery:* Fourth-year medical student

White [35] 2007–2008 Cluster (class) RCT (pilot study)	*Theoretical framework:* Theory of Planned Behaviour **Intervention—individually directed** *Format:* Educational session *Content*: Belief-based intervention (behavioural (advantages and disadvantages), normative (normative beliefs), and control (barrier and motivator) sun safe behaviours. *Duration*: 1 hour per week for 3 weeks *Delivery:* Facilitated by Cancer Council Queensland staff

^+^More detailed information and full results are provided in the Supplementary File 3.

## Data Availability

The data used to support the findings of this study are included within the article.

## Supplementary Materials

Supplementary Materials Supplementary File 1: it is referred to in Section 2.2 “Searching Literature” search strategies for systematic review. Supplementary File 2: it is referred to in Section 4.1 “Study Quality” expanded data for Table 2 (studies identified in the systematic review and reviewed). Supplementary File 3: it is referred to in Section 4.1 “Study Quality” expanded data for Table 3 (studies identified in the systematic review but not reviewed (pilot/uncontrolled)).
