# Supplementary material for: Effectiveness of Sun Protection Interventions Delivered to Adolescents in a Secondary School Setting: A Systematic Review
Source: J Skin Cancer. 2021 Mar 4;2021:6625761. doi: 10.1155/2021/6625761 (PMC7952177; doi:10.1155/2021/6625761)

**Supplementary File 1: Search strategies for Systematic review**

Cochrane

(adoles* OR teen* or middle-school) AND school AND (melanoma OR sun* OR skin* OR ultraviolet OR UV OR tann* OR nevus)

Embase


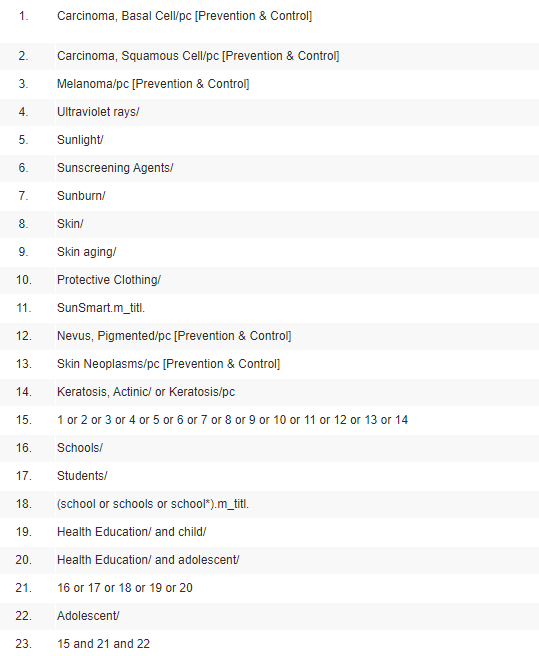


CINAHL


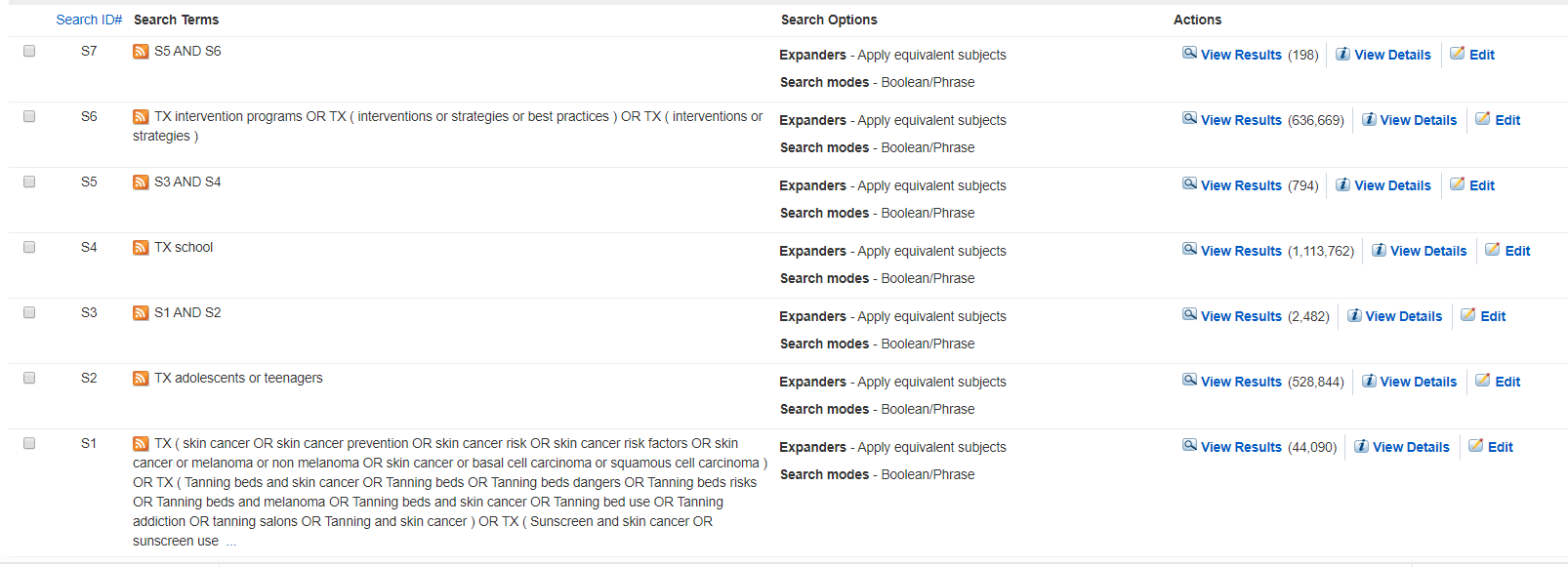


Scopus

((TITLE (adolesc* OR teen* OR middle-school) AND TITLE (school*) AND

TITLE (melanoma OR sun* OR ultraviolet OR uv OR tann* OR skin* OR nevus)) OR (( KEY (adolesc* OR teen* iddle-school) AND KEY (school*)

AND KEY (melanoma OR sun* OR ultraviolet OR uv OR tann* OR skin* OR nevus))) AND TITLE-ABS-KEY (intervent* OR program* OR educat*)

Medline (Ovid)


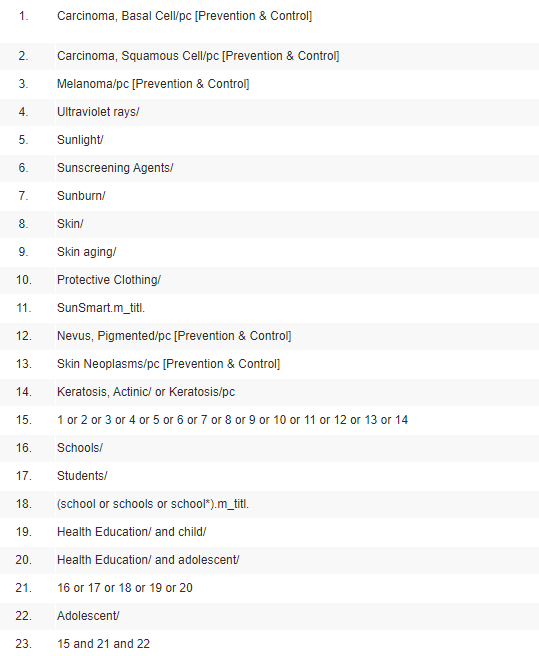


PsycInfo (Ovid)


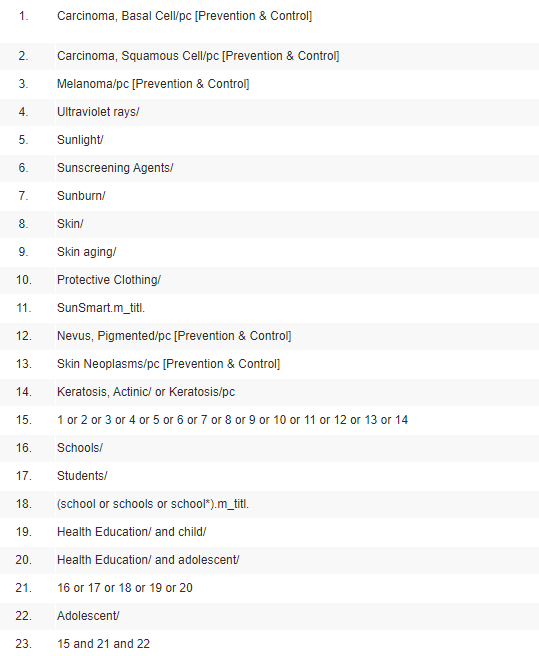


Web of Science


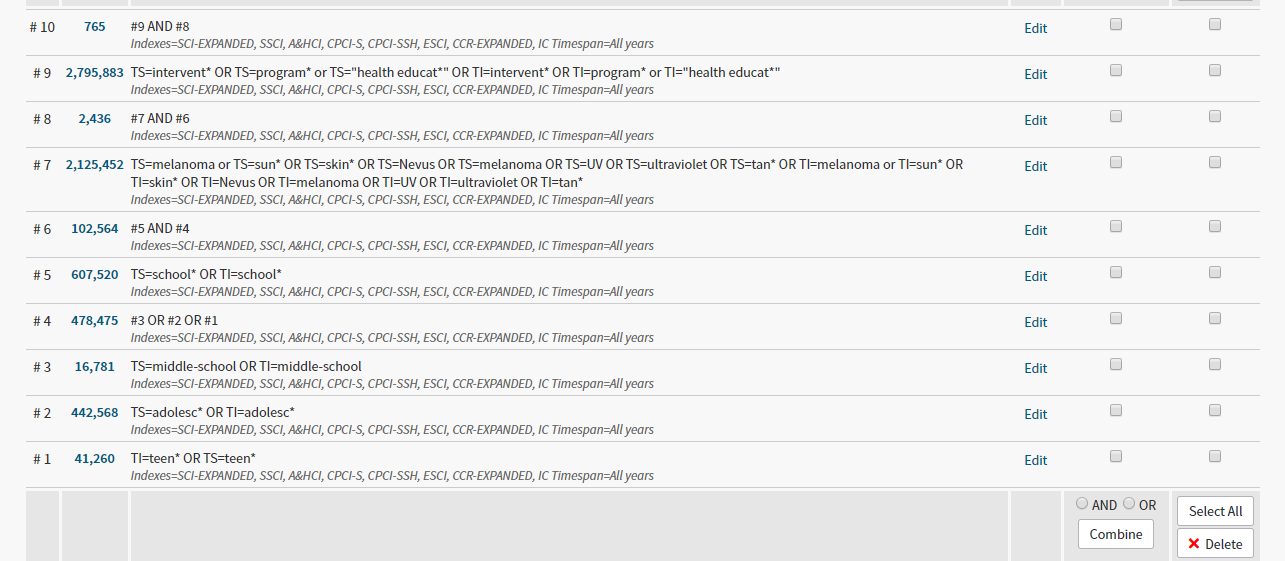

Supplement: Supplementary Materials — Supplementary File 1: it is referred to in Section 2.2 “Searching Literature” search strategies for systematic review. Supplementary File 2: it is referred to in Section 4.1 “Study Quality” expanded data for Table 2 (studies identified in the systematic review and reviewed). Supplementary File 3: it is referred to in Section 4.1 “Study Quality” expanded data for Table 3 (studies identified in the systematic review but not reviewed (pilot/uncontrolled)). [file 6625761.f1.zip › 6625761.f1/J skin cancer supplementary file 1_10 feb 2021.docx]
