# Supplementary material for: Effectiveness of Sun Protection Interventions Delivered to Adolescents in a Secondary School Setting: A Systematic Review
Source: J Skin Cancer. 2021 Mar 4;2021:6625761. doi: 10.1155/2021/6625761 (PMC7952177; doi:10.1155/2021/6625761)
Supplement: Supplementary Materials — Supplementary File 1: it is referred to in Section 2.2 “Searching Literature” search strategies for systematic review. Supplementary File 2: it is referred to in Section 4.1 “Study Quality” expanded data for Table 2 (studies identified in the systematic review and reviewed). Supplementary File 3: it is referred to in Section 4.1 “Study Quality” expanded data for Table 3 (studies identified in the systematic review but not reviewed (pilot/uncontrolled)). [file 6625761.f1.zip › 6625761.f1/J skin cancer supplementary file 2_10 feb 2021.docx]

**Supplementary File 2: Expanded data for Table 2 - Studies identified in the systematic review and reviewed**

| **Author**  **Data collection period**  **Study design**  **Global rating (EPHPP)** | **Target population**  **Sampling frame**  **Sample population** (IG=intervention group, CG=control group)  **Demographic**  **characteristics** | **Theoretical framework**  **Intervention**  **Control** | **Follow-up** BL=baseline, I=Intervention  FU=follow-up | **Results**  BL=baseline,  FU=follow-up  Y=yes N=no  *significant  IG=intervention group  CG=control group |
| --- | --- | --- | --- | --- |
| Baghianimoghadam^13^  2009  Cluster RCT  **Moderate** | *Target population:* High school students in Yazd province, Iran  *Sampling frame:* List of high schools in Department of Education of Yazd province  *Sample population:*  **Schools**  4 schools (cluster sampling and randomly assigned)  *2 schools – IG  *2 schools – CG  **Students**  360 students (180 in each group – no reported loss to follow-up)  *Demographic characteristics:*  Age (years): Mean 16.04 (0.98)  Sex: Female - 100%  Ethnicity/skin colour: Not provided | *Theoretical framework:* Protection Motivation Theory  **Intervention – individually directed**  *Format:* Lecture, group teaching and performance  *Content:* Education (not further described)  *Duration*: 3X 1 hour sessions  *Delivery:* Researchers  **Control**  Wait listed intervention | 2 month follow-up  BL: 2009  I: 2009  FU: post intervention  FU: 2 months | **Behaviour (self-report)**   \|  \|  \| BL \| FU \| \| *p value (within group)* \| \| \| \| --- \| --- \| --- \| --- \| --- \| --- \| --- \| --- \| \| *Behaviour score^1^ \| \| \| \| \| \| \|  \| \|  \| IG \| 3.92 \| 6.47 \| \| *0.001* \| \|  \| \|  \| CG \| 4.04 \| 3.88 \| \| *0.090* \| \|  \| \| *P value (between groups)* \| \| *0.571* \| *0.001* \| \|  \| \|  \| \|  \|  \|  \|  \| \|  \| \|  \| \|  \| FU (%) \| *p value (between groups)* \| \| \| \| \|  \| \| *Sunscreen \|  \|  \|  \| \|  \| \|  \| \| IG \| 68.9 \| 0.02 \| \|  \| \|  \| \| \|  \| \| CG \| 45.0 \|  \| \|  \| \|  \| \| \|  \| \| *Sunglasses \|  \|  \| \|  \| \|  \| \| \|  \| \| IG \| 26.1 \| 0.001 \| \|  \| \|  \| \| \|  \| \| CG \| 11.8 \|  \| \|  \| \|  \| \| \|  \| \| *Gloves \|  \|  \| \|  \| \|  \| \| \|  \| \| IG \| 13.3 \| 0.001 \| \|  \| \|  \| \| \|  \| \| CG \| 1.8 \|  \| \|  \| \|  \| \| \|  \| \| *Hat \|  \|  \| \|  \| \|  \| \| \|  \| \| IG \| 25.0 \| 0.001 \| \|  \| \|  \| \| \|  \| \| CG \| 7.1 \|  \| \|  \| \|  \| \| \|  \| \| *Clothes \|  \|  \| \|  \| \|  \| \| \|  \| \| IG \| 30.0 \| 0.001 \| \|  \| \|  \| \| \|  \| \| CG \| 14.8 \|  \| \|  \| \|  \| \| \|  \|   ^1^Mean Behaviour Score (summed score 9 questions (not provided) on 5 point Likert scale (anchored 5=strongly agree to 1=strongly disagree), range 0-12) |
| Brinkler^14^  2018  Cluster RCT  **Moderate** | *Target population:* High school students in Itauna, southeast Brazil  *Sampling frame:* 8 public secondary schools (number responding to invitation, total number of schools invited not provided)  *Sample population*:  **Schools**  52 school classes (cluster randomised, stratified by grade) grades 9-12  *24 IG  *28 CG  **Students**  *734 IG (46.7% of eligible)  *839 CG (53.3% of eligible)  *Demographic characteristics:*  Age (years): Mean 15.9 (1.3)  Sex: Female - 51.6%, Male - 48.4%  Skin type: 7.4% I or II, 34.9% III, 50.2% IV, 7.6% V | *Theoretical framework:* None mentioned  **Intervention “Sunface” - individually directed**  *Format:* Application (app) and education in classroom setting  *Content:* Adolescents’ selfies were altered by an app to show UVR effects on their future faces (taking into account skin type) and were shown in front of their class, accompanied by information about UVR protection. The app encompasses the effects of UVR on photoageing of the skin in general and the development of skin cancer.  *Duration:* 45 minutes  *Delivery:* Medical students  **Control**  No intervention received | 6 month follow-up  BL: February 2018  I: 1 week later  FU1: 3 months  FU2: 6 months | **Behaviour (self-report)**   \|  \| BL (%) \| FU2 (%) \| p value \| \| --- \| --- \| --- \| --- \| \| *Daily sunscreen use^1^ \|  \|  \|  \| \| IG \| 15.0 \| 22.9 \| *<0.001* \| \| CG \| 14.9 \| 14.5 \| 0.711 \| \| *Time X group* \|  \|  \| *<0.001* \| \|  \|  \|  \|  \| \| *Tanning^2^ \|  \|  \|  \| \| IG \| 18.8 \| 15.2 \| 0.019 \| \| CG \| 13.0 \| 13.6 \| 0.667 \| \| *Time X group* \|  \|  \| *0.040* \| \|  \|  \|  \|  \| \| ^1^Daily sunscreen use in 30 days preceding \| \| \| \| \| ^2^At least 1 tanning session in preceding 30 days \| \| \| \| \| ^+^Adjusted for clustering \|  \|  \|  \| |
| Dobbinson^15^  2004-2006  Cluster RCT  **Moderate** | *Target population*: Secondary school students in Melbourne, Australia  *Sampling frame:* Secondary schools (levels 7-12) outer metropolitan Melbourne (randomly selected) 300+ students, did not plan major changes to school grounds/buildings during study period (127 schools invited, 31 ineligible, 45 declined)  *Sample population*:  **Schools**  51 secondary schools (randomly allocated)  *IG – 26 schools  *CG – 25 schools  *Demographic characteristics:*  Not collected | *Theoretical framework:* None mentioned  **Intervention – Environmental directed**  *Format:* Environmental  *Content:* Two full sun areas in each school (1 intervention (primary) and 1 alternate site) had building shade sail structures installed (at intervention site only) for students to use during passive activities such as eating lunch (mean cost A$11,500 of shade sail and installation costs varied, maximum $22,000)  *Duration:* NA  *Delivery:* Environmental  **Control**  No intervention received | Continuous over 14 weeks  BL: 2004/2005 (spring / summer) – continuous 16 weeks  I: Autumn/winter 2005  FU: 2005/2006 (spring/summer) – continuous 14 weeks | **Behaviour (direct observation^1^)**   \| \|  \| BL (mean) \| FU (mean) \| Mean change \| *p value (time X group)* \| \| --- \| --- \| --- \| --- \| --- \| \| *Seeking shade^2^ \|  \|  \|  \|  \| \| IG \| 3.24 \| 5.87 \| 2.63 \|  \| \| CG \| 3.49 \| 3.46 \| -0.03 \|  \| \| Mean change \| -0.25 \| 2.41 \| 2.67 \| *0.011* \|  \| ^1^Aggregate mean of observations over observation period \| \| --- \| \| ^2^Seeking shade – using shade sails, at lunch time using video footage of mean number of students at set time points weekly \| \| \| --- \| --- \| --- \| --- \| --- \| --- \| --- \| --- \| --- \| --- \| --- \| --- \| --- \| --- \| --- \| --- \| --- \| --- \| --- \| --- \| --- \| --- \| --- \| --- \| --- \| --- \| --- \| --- \| \|  \| |
| Jeihooni^16^  2016-2017  Cluster RCT  **Moderate** | *Target population*: High school students in Fasa City, Fars Province, Iran  *Sampling frame:* All male high schools in the area (N=22)  *Sample population*:  **Schools**  4 schools randomly selected  *IG - 2  *CG - 2  **Students** (participation rates not reported)  *IG - 150  *CG - 150  *Demographic characteristics:*  Age (years):  IG: 16.05 (1.76)  CG: 16.20 (1.71)  Sex: 100% Male  Ethnicity/skin colour: Not provided | *Theoretical framework:* PRECEDE model  **Intervention**  *Format:* Educational session including group discussion, questions and answers, practical presentation, use of videos, PowerPoint presentation, instruction booklet.  *Content:* education - skin health, skin cancer and risks, sunlight, sun protection.  *n.b. Telephone group also organised for students parents.*  *Duration:* 6 training sessions of 45-50 minutes duration held on weekly basis  *Delivery:* Research team  **Control**  No intervention received | 4 months  BL: 2016-2017  I: 2016-2017  FU: 4 months | **Behaviour (self-report)^1^**   \|  \| BL (mean) \| FU (mean) \| *p value (within group)* \| \| \| --- \| --- \| --- \| --- \| --- \| \| IG \| 4.11 \| 11.65 \| *0.001* \|  \| \| CG \| 4.26 \| 4.70 \| *0.104* \|  \| \| *P value (between groups)* \| *0.417* \| *0.001* \|  \|  \|   ^1^Skin cancer preventive behaviours – 7 questions (actual questions not provided – 5 questions Y/N (scored 0 or 1) and 2 questions on 4 point Likert scale (scored 0-3, anchor points not provided) - summed to provided total score with range from 0-15 |
| Rahmatiasl ^17^  Year not provided  Pre/post (control group)  **Medium** | *Target population:* 1^st^ grade high school students in Ahwaz, Iran  *Sampling frame:* Education 4 District of Ahwaz city  *Sample population*:  **Schools**  4 schools (2 boys and 2 girls) randomly selected. Allocation of group non-randomised.  *IG 2 schools  *CG 2 schools  **Students** – 215 students  *IG 3 classes  *CG 3 classes  *Demographic characteristics:*  Age (years): 1^st^ grade (13 years)  Sex: 47.9% - Females, 52.1% - Males  Ethnicity/skin colour: Not provided | *Theoretical framework:* Protection Motivation Theory  **Intervention**  *Format:* Lecture and question and answer session. Pamphlet also distributed.  *Content:* UVR, UVR and health effects of exposure to UVR, the factors affecting the exposure to UV light, the importance of sun protection in childhood and adolescence, how to protect from the sunlight, benefits of using protective devices against the sunlight and correct ways to use sunscreen.  *Duration:* Not specified  *Delivery:* Not specified  **Control**  No intervention received | 4 months  BL: Prior to Intervention  I: Not specified  FU: 4 months | **Behaviour (interview administered self-report)^1^**   \|  \| BL (mean) \| FU (mean) \| *p value (within group)* \| \| --- \| --- \| --- \| --- \| \| IG \| 3.73 \| 6.44 \| *0.000* \| \| CG \| 4.43 \| 4.28 \| *0.699* \| \| *p value (between groups)* \| *0.075* \| *0.000* \|  \| \|  \|  \|  \|  \| \| ^1^10 questions (not provided) (scored 0-14 – No information provided on how this is derived) \| \| \| \| |
| Aarestrup^18^  2010-2011  Cluster RCT  **Weak** | *Target population*: 14-18 year old students in Denmark  *Sampling frame*: All continuation schools in Denmark were contacted (N=264), and those that did not meet the eligibility criteria (N=103), declined to participate (N=67), excluded for economic reasons (N=5) or withdrew (N=31) were excluded  *Sample population*:  **School** - 33 schools agreed to be randomised (48% of those eligible)  *16 IG (69% implemented)  *17 CG (100% implemented)  **Individual students**  *3,635 enrolled (2,351 (65%) completed baseline and follow-up *(note 2,323 are reported in tables)*  *Demographic characteristics:*  Age (years): 14 - 8%, 15 - 42%, 16 - 47%, 17 - 3%  Sex: Female - 51%  Ethnicity/skin colour: Not provided | *Theoretical framework:* None mentioned  **Intervention – individually directed**  *Format:* E-magazine, short films, advertisements, campaign materials, paintings, social media, poetry, fiction & literature  *Content:* Health risks associated with sunbed use as well as appearance damaging effects  *Duration:* Mean 5.6 lessons per class  *Delivery:* Classroom teacher – teachers guide provided  **Control**  No intervention received | Approximately 6 months  BL: September 2010  I: November 2010  FU: May-June 2011 | **Behaviour (self-report)**   \| Sunbed^1^ \| \|  \| \|  \| \|  \| \|  \| \|  \| \| \| --- \| --- \| --- \| --- \| --- \| --- \| --- \| --- \| --- \| --- \| --- \| --- \| \|  \|  \| \|  \| \| Odds ratio \| \|  \| \| *P value* \| \| \|  \| Females \| \|  \| \| 0.60 \| \|  \| \| *0.005* \| \| \|  \| Males \| \|  \| \| 0.58 \| \|  \| \| *0.030* \| \|  \| ^1^Sunbed use in past 6 months (Y/N) \| \| --- \| \| ^+^Adjusted baseline measures - age, gender, smoking, socioeconomic position, parental attitudes to sunbed use \| |
| Buendia-Eisman^25^  2009  Cluster RCT  **Weak** | *Target population:* Students aged 12-16 years in Andalusia, Spain  *Sampling frame:* Public secondary schools from 8 provincial capitals of Andalusia that completed a baseline survey (randomly selected 25 schools)  *Sample population*:  **Schools**  12 secondary schools (randomly selected and randomly allocated)  *IG 7 schools  *CG 5 schools  ***Students***  *IG 730 students (306 completed follow-up)  *CG 560 students (223 completed follow-up)  *Demographic characteristics:*  Age (years): 12-16, Mean 13.75  Sex: 49.8% Female, 50.2% Male  Skin type: 11.5% I or II, 76.8% III or IV, 11.8% V or VI | *Theoretical framework:* None mentioned  **Intervention “Healthy Sun Habits” - individually directed**  *Format:* Online web page  *Content:* Webpage structured  *the sun – sun & UVR characteristics, dangers of sunburn  *sun without danger – emphasised factors associated with sunburn and appropriate sun protection behaviours  *key sun protection messages  *games and website links  *Duration:* Pupils used the website for at least 1 hour at school and then had it available for use through the summer.  *Delivery:* Entirely on the internet with teachers only providing technical support for using webpage.  **Control**  No intervention received | 3 months  BL: May 2009  I: June 2009  FU: October 2009 | **Physiological measures (self-report)**   \|  \| Odds ratio^+^ \| *p value* \| \| --- \| --- \| --- \| \| ***Sunburn^1^ \| 0.45 \| *0.018* \| \| ***Sunburn with blisters^2^ \|  \| *NS* \|  \| ^1^Presence of sunburn (moderate degree of painful erythema lasting a few days) previous summer (Y/N) \| \| --- \| \| ^2^Presence of sunburn with blisters previous summer (Y/N) \| \| +adjusted for pre-intervention response variables, intervention group variable, gender, inland/coastal centre \|   **Behaviour (self-report)**   \|  \| Odds ratio+ \| *p value* \|  \| \| --- \| --- \| --- \| --- \| \| ***Sunbathing (never/sometimes)^3^ \| 0.317/0.909 \| *0.169* \|  \| \| ***Sun protection^4^ \| 0.949 \| *0.757* \|  \| \| ***Sun protection – cloudy^5^ \| 1.318 \| *0.041* \|  \| \| ***Sun cream^6^ \| 0.045 \| *0.05* \| \| \| ***Sun cream (never/sometimes)^7^ \| 0.787/1.498 \| *0.05* \|  \|  \| ^3^ Sunbathing between midday and 6pm (5 point Likert scale collapsed – (never, almost never), (sometimes), (almost always, always=reference group)) previous summer \| \| --- \| \| ^4^ Sun protection measures; ^5^sun protection measures when cloudy, ^6^suncream (Y/N) \| \| ^7^Sun cream (5 point Likert scale collapsed – (never, almost never=never), (sometimes), (almost always, always=reference group)) previous summer \| |
| Lai^20^  2012-2014  Cluster RCT  **Weak** | *Target population:* High schools students aged 12-18 years in Beijing, China  *Sample population*:  **Schools**  Three high schools (convenience sample)  **Students** (random cluster sampling, classroom=unit)  *IG1 – 209 students  *IG2 – 218 students  *CG – 211 students  *Demographic characteristics:*  Age (years): Mean 14.4 (2.5)  Sex: 51% - Female, 49% - Male  Ethnicity/skin colour: Not provided | *Theoretical framework:* None specified  **Intervention**  *IG1*  *Format:* Presentations, photograph and pamphlets  *Content:*  ***Education *–* nature and dangers of UVR, sun protection methods, correct use of sunscreen  *A photograph was taken of students to assess skin type and students taught how to protect themselves according to skin type and UV index  *Pamphlets contained highlights of presentation  *Duration:* 2 X 45 minutes per year (over 3 years)  *Delivery:* Received 2X during year 1 and year 2  *IG2*  *Content:* Educational pamphlet (as IG1)  *Format:* Pamphlet  *Duration:* Received 2X during year 1  *Delivery:* NA  **Control**  No intervention received | 2 years  BL: May 2012  I: Following baseline  FU1: 1 year later  FU2: 2 years later | \| **Behaviour (self-report)** \| \| \| \| \| \| \| \| \| --- \| --- \| --- \| --- \| --- \| --- \| --- \| --- \| \|  \| BL (%) \| FU1 (%) \| FU2 (%) \| *P value* \| \|  \| \| \| *Sunscreen \|  \|  \|  \|  \|  \| \| \| IG1: \| 54.1 \| 53.6 \| 81.3 \| *<0.001* \| (FU2 compared with BL) \| \| \| IG2: \| 49.1 \| 49.1 \|  \|  \|  \| \| \| CG: \| 45.0 \| 41.2 \|  \|  \|  \| \| \| *Protective clothes \| \| \| \| \|  \| \| \| IG1: \| 30.1 \| 24.9 \| 62.2 \| *<0.001* \| (FU2 compared with BL) \| \| \| IG2: \| 25.7 \| 26.1 \|  \|  \|  \| \| \| CG: \| 20.4 \| 21.8 \|  \|  \|  \| \| \| *Hats \|  \|  \|  \|  \|  \| \| \| IG1: \| 31.1 \| 35.4 \| 67.0 \| *<0.001* \| (FU2 compared with BL) \| \| \| IG2: \| 25.2 \| 31.2 \|  \|  \| \|  \| \| \| CG: \| 26.5 \| 23.2 \|  \|  \| \|  \| \| \| *Sun umbrella \| \| \| \| \| \|  \| \| \| IG1: \| 32.5 \| 33.5 \| 63.6 \| *<0.001* \| \| (FU1,FU2 compared with BL) \| \| \| IG2: \| 32.1 \| 28.4 \|  \|  \| \|  \| \| \| CG: \| 28.4 \| 25.6 \|  \|  \| \|  \| \| \| *Sunglasses \|  \|  \|  \|  \| \|  \| \| \| IG1: \| 32.6 \| 26.3 \| 20.1 \| *NS* \| \|  \| \| \| IG2: \| 19.3 \| 22.5 \|  \|  \| \|  \| \| \| CG: \| 21.3 \| 16.6 \|  \|  \| \|  \| \| \| *Avoiding sun exposure at school \| \| \| \| \| \| \| \| \| IG1: \| 18.7 \| 35.4 \| 67.0 \| *<0.001* \| \| (FU2 compared with BL) \| \| \| IG2: \| 19.3 \| 31.2 \|  \|  \| \|  \| \| \| CG: \| 23.7 \| 23.2 \|  \|  \| \|  \| \| \| *Seeking shade as far as possible \| \| \| \| \| \| \| \| \| IG1: \| 27.8 \| 38.3 \| 63.6 \| *<0.001* \| \| (FU1,FU2 compared with BL) \| \| \| IG2: \| 23.4 \| 34.4 \|  \|  \| \|  \| \| \| CG: \| 28.4 \| 25.6 \|  \|  \| \|  \| \| \|  \|  \|  \|  \|  \| \|  \| \| \| **Physiological outcomes (self-reported)** \| \| \| \| \| \| \| \| \| *Sunburn^1^ \|  \|  \|  \|  \| \|  \| \| \| IG1: \| 48.3 \| 30.1 \| 15.8 \| <0.001 \| \| (FU1,FU2 compared with BL) \| \| \| IG2: \| 42.2 \| 31.2 \|  \|  \| \|  \| \| \| CG: \| 32.7 \| 28.4 \|  \|  \| \|  \| \| \| *Suntan^2^ \|  \|  \|  \|  \| \|  \| \| \| IG1: \| 78.9 \| 58.9 \| 42.6 \| <0.001 \| \| (FU1 compared with BL) \| \| \| IG2: \| 77.1 \| 62.4 \|  \|  \| \|  \| \| \| CG: \| 74.4 \| 67.3 \|  \|  \| \|  \| \| \|  \|  \|  \|  \|  \| \|  \| \| \| ^1^Suffered from sunburn in recent 12 months \| \| \| \| \| \| \| \| \| ^2^Ever had suntan in most recent 12 months \| \| \| \| \| \| \| \| |
| Lowe^12^  1993-1995  Pair RCT  **Weak** | *Target population:* Grade 8 high school students (moving onto grade 9 and 10) in Queensland, Australia  *Sample population*:  **Schools**  All public secondary schools Grades 8-10 in two Queensland Education Regions (n=26) Pair matched and randomised to group:  *IG – 13 schools  *CG – 13 schools  **Students**  *3400 students  *IG 1,754 students  *CG 1,976 students  *Demographic characteristics:*  Age (years): Grade 8 (13 years)  Sex: Not provided  Ethnicity/skin colour: Not provided | *Theoretical framework:* based on behavioural theory that emphasize the importance of social and environmental influences, and behavioural strategies  **Intervention**  *Format:* Participatory learning principals active student involvement including role playing, problem solving and student directed activities  *Content:*  *Module content varied by grade level. Issues covered related to the need to protect yourself from the sun, behavioural strategies related to using sun protection measures, personal and social images of having a tan, use of sun safe clothing, and how to change their schools through structural change.  *Schools asked to adopt a school policy document  *Duration:* 3 modules delivered sequentially to students as they moved through grades 8,9 and 10. Each module contained at least 4X 50 minute classroom lessons with 2 optional extension lessons. Six week program  *Delivery:* Health and physical activity teacher (trained in use of module)  **Control**  Received usual health education provided by teachers, no restrictions placed on other programs related to sun exposure | 3 years  Round 1  Pre1: November 1993  I: November 1993 (just prior to summer break)  FU1: February 1994  Round 2  Pre2: November 1994  I: November 1994 (just prior to summer break)  FU2: February 1995  Round 3  Note: no pre-survey  I: November 1995 (just prior to summer break)  FU3: February 1996 | **Behaviour (self-report)**   \|  \| Pre1  (mean) \| FU2  (mean) \| Pre2  (mean) \| FU2  (mean) \| FU3  (mean) \| *p value^+^* \| \| --- \| --- \| --- \| --- \| --- \| --- \| --- \| \| *SPBI^1^ (Sunday) \|  \|  \|  \|  \|  \|  \| \| IG \| 55.29 \| 57.63 \| 55.67 \| 54.39 \| 54.01 \|  \| \| CG \| 55.34 \| 54.62 \| 55.87 \| 55.00 \| 54.55 \|  \| \| *Time X group* \|  \|  \|  \|  \|  \| *0.044* \| \|  \|  \|  \|  \|  \|  \|  \| \| *SPBI^1^ (Monday) \|  \|  \|  \|  \|  \|  \| \| IG \| 49.77 \| 52.90 \| 50.77 \| 51.53 \| 52.58 \|  \| \| CG \| 50.42 \| 51.56 \| 51.14 \| 51.45 \| 51.64 \|  \| \| *Time X group* \|  \|  \|  \|  \|  \| *0.225* \|  \| ^1^Sun protection behaviours index (SPBI) **-** summarised sun protection behaviour exhibited during periods outdoor (weighted by URV levels) – aggregated (range 1-100) \| \| --- \| \| + adjusted for pairing effects in ANOVA model \| |
| Sumen^21^  2013  Non randomised trial (with control group)  **Weak** | *Target population:* Maritime high school students in Antalya, Turkey  *Sampling frame*: Maritime high schools  *Sample population*:  **Schools**  2 schools (convenience sample)  **Students**  *IG 389  *CG 178  *Demographic characteristics:*  Age (years): 14 - 2.6%, 15 - 25.7%, 16 - 31.7%, 17 - 25.4%, 18 - 14.6%  Sex: 10.1% Female, 89.9% Male  Ethnicity/skin colour: Not provided | *Theoretical framework:* None specified  **Intervention**  *Format:* Didactic (classroom), brochures (take home) and posters (environment)  *Content:* Training regarding skin cancer, sun protection steps and harmful effects of the sun followed by “Dear 16-year old me” video which emphasises the importance of sun protection in the adolescent period. Educational material also provided to students at end of training session. Four weeks following training posters were hung within the school and classrooms as a reminder.  *Duration:* 35-45 minutes  *Delivery:* Not specified  **Control**  Wait listed intervention | 3 months  BL: March 2013  I:Following BL  FU: June 2013 | **Behaviour (self-report)**   \|  \| BL (%) \| FU (%) \| *p value*  *(within group)* \| \| --- \| --- \| --- \| --- \| \| *SP cream^1^ \|  \|  \|  \| \| IG \| 76.1 \| 86.1 \| *0.000* \| \| CG \| 55.6 \| 60.7 \| *0.417* \| \| *SP cream – beach^2^ \|  \|  \|  \| \| IG \| 41.4 \| 23.9 \| *0.000* \| \| CG \| 53.9 \| 49.4 \| *0.466* \| \| *SP cream – long time^3^ \|  \|  \|  \| \| IG \| 49.9 \| 65.0 \| *0.000* \| \| CG \| 33.1 \| 44.9 \| *0.036* \| \| *SPF below 20^4^ \|  \|  \|  \| \| IG \| 45.2 \| 18.2 \| *0.000* \| \| CG \| 53.7 \| 51.1 \| *0.664* \| \| *Remain in shade^5^ \|  \|  \|  \| \| IG \| 42.4 \| 59.6 \| *0.000* \| \| CG \| 50.6 \| 55.6 \| *0.402* \| \| *Stay indoors^6^ \|  \|  \|  \| \| IG \| 82.0 \| 90.2 \| *0.001* \| \| CG \| 74.2 \| 78.7 \| *0.366* \| \| *Clothing - shoulders^7^ \|  \|  \|  \| \| IG \| 50.6 \| 56.6 \| *0.106* \| \| CG \| 60.7 \| 63.5 \| *0.678* \| \| *Sunglasses^8^ \|  \|  \|  \| \| IG \| 66.6 \| 77.6 \| *0.001* \| \| CG \| 56.2 \| 60.7 \| *0.461* \| \| *SP hats^9^ \|  \|  \|  \| \| IG \| 54.2 \| 60.9 \| *0.064* \| \| CG \| 52.8 \| 57.3 \| *0.470* \| \| \| ^1^Use of sun protection cream (Y/N) \| \| --- \| \| ^2^Use of sun protection cream while going out at the beach etc. (Y/N) \| \| ^3^Use of sun protection creams, repeatedly when under the sun for long periods of time (Y/N) \| \| ^4^Protection factors of the sun cream that is used (SPF below 20/ SPF above 20) \| \| ^5^Preferred places in summer time as far as possible (shade/sun) \| \| ^6^Paying attention not to go out between the hours 10:00-16:00 (Y/N) \| \| ^7^Generally preferred type of t-shirt in summer months (cover shoulders/exposure shoulders) \| \| ^8^Use of sunglasses while going out (Y/N) \| \| ^9^Use of hats with long edges when going out (Y/N) \| \| \| \| \| \| \| \|  \| \| \| \| \| |
| Tuong^22^  2012  RCT  **Weak** | *Target population:* 11 grade students (aged 16-17 years) in California, USA  *Sample population*:  **School**  One school (convenience sample)  **Students**  *IG 25 students  *CG 25 students  *Demographic characteristics:*  Age (years):  *IG: Mean age 17.1 (0.88)  *CG: Mean age 17.2 (0.44)  Sex: IG: 76% Female, 24% Male  CG:84% Female, 16% Male  Skin type: IG: 12% White, 88% non-White  CG: 4% White, 96% non-white | *Theoretical framework:* Health Belief Model  **Intervention**  *Format:* Integrated into the classroom health education, viewed video assigned as a group  *Content:* Appearance-based video on UV induced premature ageing  *Duration:* video 5 minute duration  *Delivery:* Assume classroom teacher  **Control**  Health based video emphasizing UV exposure and skin cancer risk | 6 weeks  I: February to March 2012  FU: 6 weeks | **Behaviour (self-report)**   \|  \| BL (mean) \| FU (mean) \| *p-value (within group)* \| \| --- \| --- \| --- \| --- \| \| *Sunscreen adherence (days/week) \| \|  \|  \| \| IG \| 0.6 \| 2.8 \| *<0.001* \| \| CG \| 0.7 \| 0.9 \| *0.0096* \| \| *p value (between groups)* \| *0.792* \| *0.003* \|  \| \| *Shade^1^ (never/rarely ) \| \| \|  \| \|  \| BL (%) \| FU (%) \|  \| \| IG \| 20 \| 20 \| *1.000* \| \| CG \| 8 \| 12 \| *1.000* \| \| *p value (between groups)* \| *0.417* \| *0.702* \|  \| \| *Hat^2^ (never/rarely %) \| \|  \|  \| \| IG \| 92 \| 80 \| *0.453* \| \| CG \| 84 \| 76 \| *0.727* \| \| *p value (between groups)* \| *0.667* \| *1.000* \|  \| \| *Long sleeved shirt^3^ (never/rarely %) \| \| \|  \| \| IG \| 76 \| 64 \| *0.375* \| \| CG \| 88 \| 88 \| *1.000* \| \| *p value (between groups)* \| *0.463* \| *0.095* \|  \| \|  \|  \|  \|  \| \| ^1^How often do you stay in the shade when out in the sun for > 1 hour? \| \| \| \| \| ^2^How often do you wear a hat that shades your face, ears, and neck when out in the sun >1 hour? \| \| \| \| \| ^3^How often do you wear a long-sleeved shirt when out in the sun for >1 hours? \| \| \| \| |
| White^23^  Date not provided  Cluster RCT  **Weak** | *Sampling frame:* High school students (aged 12-17 years) in Queensland, Australia  *Sample population*:  **Schools – randomly allocated**  9 schools (public and private)  **Students**  382 students - (analysed 213)  *Demographic characteristics:*  Age (years): Mean 13.73  Sex: 61.1% Female 38.9% Male  Skin type: 59% Very fair or fair | *Theoretical framework:* Theory of planned behaviour  **Intervention**  *Format:* Group based discussions, role playing and goal setting.  *Content:* Session 1: sun protection related attitudes and beliefs, long/short term effects of sun exposure and advantages and disadvantages of sun protection  Session 2: Foster perceptions of normative beliefs on sun protection.  Session 3: Aimed to increased perceptions of self-efficacy over using sun protection measures  *Duration:* 1 hour per week for 3 weeks  *Delivery:* Facilitated by Cancer Council Queensland staff  **Control**  Wait listed intervention | 4 week follow-up  BL: Date not provided  FU1: 1 week  FU2: 4 week | **Behaviour (self-report)**   \|  \| BL \| FU2 \| *p value (time X group)* \| \| --- \| --- \| --- \| --- \| \| *Sun safe behaviour^1^ \|  \|  \|  \| \| ***Weekday*** \| 2.75 \| 3.19 \| *NS* \| \| IG \| 2.91 \| 3.40 \|  \| \| CG \|  \|  \|  \| \| ***Weekend*** \|  \|  \|  \| \| IG \| 3.19 \| 3.75 \| *0.043* \| \| CG \| 3.32 \| 3.37 \|  \| \|  \|  \|  \|  \| \| ^1^Not defined, weighted \| \| \| \| |
| Wu^24^  2017  Cluster (school) non randomised trial (with control group)  **Weak** | *Target population:* High school students grades 9-12 (14-18 years) in Utah, USA  *Sampling frame:* 13 schools  *Sample population*:  **Schools**  11 schools agreed to participate – assigned at this level  **Students**  1,573 students (735 have baseline measures - 38% of targeted body included in the study)  *Demographic characteristics:*  Age (years): 9^th^ grade - 26.2%, 10^th^ grade 52.3%, 11^th^ grade – 12.1%, 12^th^ grade - 9.3%  Sex: 49.5% Female, 50% Male, 0.5% other  Race: 62.5% non-Hispanic White, 25.8% Hispanic, 2.8% African American, 2.0% American Indian, 3.2% Asian American, 3.7% Other | *Theoretical framework:* Extended parallel process model – communicate health risk and prevention information by targeted individual’s perceived threat and perceived efficacy.  **Intervention**  **IG1**  *Format:* Health or science class, PowerPoint and interactive activity (3-4 students per group), classroom discussion  *Content:* Education (see CG) plus a sunscreen activity which illustrates the UVR blocking properties of sunscreen of differing SPF levels  *Duration:* 1 classroom period  *Delivery:* Research assistants  **IG2**  *Format:* Health/science class  *Content:* Education (see CG) and receipt of a printed personalised photograph showing current skin damage cause of UVR exposure. Class discussion on how these photos related to UVR damage and skin cancer risk.  *Duration:* 1 classroom period  *Delivery:* Research assistants  **IG3**  *Format:* Health/science class, PowerPointand interactive activity (individual)  *Content:* Education (see CG) and behavioural change worksheet aimed to improve self-efficacy using sun protection goal setting and planning. Students first selected a behaviour they were willing to commit to implementing in the next month  *Duration:* 1 classroom period  *Delivery:* Research assistants  **Control**  *Format:* Health/science class – PowerPointand interactive activity (individual), classroom discussion  *Content:* Skin cancer education – incidence, risk factors, causes, strategies to prevent and screen, common misconceptions and prevention strategies  *Duration:* 1 classroom period  *Delivery:* Research Assistants | 1 month  BL: March-May 2017  I: Not stated  FU: 1 month | **Behaviour (self-report)**  Survey instrument – adapted from Sun Habits survey (reference provided)  *Behaviour^1^*  *Sunscreen application (Means)  BL FU *p value (within group)*  IG1 2.17 2.90 *0.001*  IG2 1.99 2.28 *0.009*  IG3 1.87 3.55 *<0.001*  CG 2.03 2.60 *0.001*  *Sunscreen reapplication  IG1 1.80 2.54 *0.001*  IG2 1.69 2.14 *0.002*  IG3 1.55 3.29 *<0.001*  CG 1.77 2.30 *0.002*  *Long sleeved shirt  IG1 2.68 2.87 *0.01*  IG2 2.92 3.04 *0.03*  IG3 2.77 3.80 *0.003*  CG 2.78 3.12 *0.01*  *Long pants or skirt  IG1 3.40 2.84 *0.006*  IG2 3.63 3.27 *0.11*  IG3 3.60 2.69 *0.002*  CG 3.41 3.29 *0.291*  *Wide brimmed hat  IG1 1.75 2.30 *<0.001*  IG2 1.83 1.99 *0.17*  IG3 1.90 3.11 <0.001  CG 1.91 2.31 0.02  *Shade or umbrella  IG1 2.62 3.18 *<0.001*  IG2 2.48 2.71 *0.01*  IG3 2.31 3.78 *0.001*  CG 2.48 3.08 *0.003*  *Avoid peak hours  IG1 2.13 2.81 *<0.001*  IG2 2.11 2.44 *0.004*  IG3 1.99 3.56 *<0.001*  CG 2,14 2.78 *0.002*  *Sunglasses  IG1 2.60 3.17 *0.01*  IG2 2.47 2.83 *0.06*  IG3 2.42 3.73 *0.003*  CG 2.46 2.94 *0.04*  Intentional tanning  *Outdoor intentional tanning  IG1 2.13 2.78 *0.001*  IG2 1.95 2.16 *0.047*  IG3 2.27 3.60 *0.002*  CG 2.10 2.52 *0.008*  *Indoor tanning  IG1 1.09 1.44 *<0.001*  IG2 1.12 1.34 *0.001*  IG3 1.17 1.90 *<0.001*  CG 1.17 1.45 *<0.001*  **Physiological outcomes**  *Sunburn^2^  IG1 1.35 2.20 *<0.001*  IG2 1.25 1.83 *<0.001*  IG3 1.20 3.12 *<0.001*  CG 1.35 2.05 *<0.001*  ^1^How often in the past month did you engage in sun protection and tanning behaviours? (5 point Likert scale anchored at 1 (never) to 5 (always).  ^2^ Red or painful and lasted a day or more (BL=12 months, FU=past month) |
