# Supplementary material for: Effectiveness of Sun Protection Interventions Delivered to Adolescents in a Secondary School Setting: A Systematic Review
Source: J Skin Cancer. 2021 Mar 4;2021:6625761. doi: 10.1155/2021/6625761 (PMC7952177; doi:10.1155/2021/6625761)
Supplement: Supplementary Materials — Supplementary File 1: it is referred to in Section 2.2 “Searching Literature” search strategies for systematic review. Supplementary File 2: it is referred to in Section 4.1 “Study Quality” expanded data for Table 2 (studies identified in the systematic review and reviewed). Supplementary File 3: it is referred to in Section 4.1 “Study Quality” expanded data for Table 3 (studies identified in the systematic review but not reviewed (pilot/uncontrolled)). [file 6625761.f1.zip › 6625761.f1/J skin cancer supplementary file 3_10 feb 2021.docx]

**Supplementary File 3: Expanded data for Table 3 - Studies identified in the systematic review but not reviewed (pilot/uncontrolled)**

| **Author**  **Data collection period**  **Study design** | **Target population**  **Sampling frame**  **Sample population** (IG=intervention group, CG=control group)  **Demographic characteristics** | **Theoretical framework**  **Intervention**  **Control** | **Follow-up** |
| --- | --- | --- | --- |
| Davis^41^  2010  Pre/post (uncontrolled) (pilot study) | *Target population:* high school students in Tuscan, Arizona  *Sample population*:  **Schools**  4 high schools in 5 districts of Arizona (note also included 5 middle schools but some results reported separately) (convenience sample)  **Students**  249 students (provides composite numbers for follow-up, not disaggregated for high school students)  *Demographic characteristics:*  No demographic information collected | *Theoretical framework:* Health Belief Model – specifically cue to action and increase self-efficacy  **Intervention – “Project Students are SunSafe”**  *Format:* Power point presentation and three interactive activities in classroom setting  *Content:*  Education - Epidemiology, skin structure, skin cancer types, UVR, sun protection strategies, tanning consequences.  *Perceived susceptibility – video testimonial of 11 year melanoma survivor  *Perceived severity of UVR skin damage - images of overexposed individuals are presented (including celebrities)  *Benefits of not engaging in indoor tanning – displaying items that could be bought in exchange for money not spend in tanning booths  Interactive activities – students shown skin through filtered UVR analyser, sunscreen, UVR detecting Frisbee used to visualise protection provided by different fabrics  *Duration:* 50-65 minutes (25 minutes of this is Powerpoint)  *Delivery:* University students (trained in sun safety and presentation)  **No control** | 2-4 weeks |
| Geller^42^  2001  Pre/post (pilot study) | *Target population:* 10^th^ grade students in Palm Beach County, Florida  *Sample population*  **School**  1 School (convenience sample)  **Students**  344 Students enrolled (184 completed follow up)  *Demographic characteristics:*  Age (years): Mean 16.3  Sex: 57% Female, 43% Male  Skin type: 48.1% White, 23.1% Latino, 20.3% African American, 7.1% Asian, Other 1.0% | *Theoretical framework:* “Theory driven” exact theory not specified  **Intervention “SunSmart America”**  *Format:* Education - Science (biology) classes  *Content:* cancer prevention and detection curriculum integrated into school biology classes (based on SunSmart Australia curriculum for Victorian schools in Australia) - What is Cancer, Types of Cancer, Are you SunSmart? The Genetics of Skin Cancer, Sunburn and the UV Index, Natural Selection and Skin Colour, SunSmart Health habits  *Duration:* Choose 7 of 12 modules – 45-60 minutes in duration  *Delivery:* Classroom teacher  **No control** | 6 months |
| Hughes^43^  1990  Post intervention (controlled) | *Target population:* High school students in England  *Sample population*: 7 schools (convenience sample)  *Target population*: 543 students  *Demographic characteristics:*  Age (years): 12-13 - 22%, 14 - 46%, 15 - 19%, 16+ - 12%, Do not know 1%  Sex: 48% Female, 51% Male, Missing 1%  Ethnicity/skin colour: Very fair 8%, Fair 30%, Medium 42%, Olive 8%, Dark 7%, Very dark 1%, Black 1%, Do not know 3% | *Theoretical framework:* None specified  **Intervention**  *Format:* Leaflet. workbook and video  IG1 – education + workbook  IG2 – education + workbook + video  IG3 – education + workbook + homework to design posters  IG4 – education + additional discussion later in the week  *Content:* education modules in health and physical education classes delivered. Materials: i). Colour leaflet designed to make covering up look cool ii). Workbook contained information on sun, UVR and skin cancer, iii). Video – celebrity discusses concepts of sun and skin cancer with class of children.  *Duration:* No information  *Delivery:* Classroom – research team  **Control**  No intervention received | 4 months |
| Kouzes^44^  2015-2016  Pre/post (Uncontrolled) (pilot) | *Target population:* School students grade 4 to 10 in Nevada, USA (Note:. results separated out for high school students)  *Sample population*:  **Schools**  7 Nevada schools (convenience sample)  **Students** (grade 10 reported separately)  *BL:88 pre intervention survey  *FU:102 post intervention survey  (not linked)  *Demographic characteristics:*  Age (years): 10^th^ grade (15-16)  Sex: No provided  Ethnicity/skin colour: Not provided | *Theoretical framework:* None specified  **Intervention “SunSmart Schools pilot program”**  *Format:* Curriculum delivered in health class, environmental – provision of sunscreen  *Content:*  *Schools asked to implement a written sun protection policy.  School given flexibility with which aspects of the program to implement, options included:  *Age relevant evidence based curriculum materials- evidence based, easy to use and teach and met schools common core requirements  *Daily UV index announcements  *Guest speaker  *Provision of sunscreen  *Allowing students to wear hats and sunglasses.  *Duration:* Not specified  *Delivery:* School staff  **No control** | Approximately 8 months |
| Loescher^45^  December 2016-March 2017  Pre/post (uncontrolled) | *Target population*: Hispanic/Latino high school students in Rural south eastern Arizona, USA  *Sample population*:  **School**  3 high schools (convenience sample)  **Students**  198 students analysed (90% of those at BL)  *Demographic characteristics:*  Age (years): 15.07 ± 1.07  Sex: 65.5% - Female, 34.5% - Male  Skin type: 95.5% Hispanic or Latino, 4.5% non-Latino | *Theoretical framework:* None specified  **Intervention “Project Students are SunSafe” (adapted for Hispanic/Latino students)”**  *Format:* Classroom lesson  *Content:*  *Education - PowerPoint presentation – included information on sunscreen ingredients  *Three activities – interactive sun protective fabric. Sunscreen ingredient or skin analyser activity  *Duration:* 40 minutes (3 modules took no longer than 20 minutes to deliver)  *Delivery:* Students from school were trained as peer educators to implement the lesson (in groups of 3) to their peers at their school  **No control** | 3 months |
| Milijkovic^46^  2007/2008 (2 periods of data collection)  Pre/post (uncontrolled) | *Target population*: High school students – 1^st^ and 2^nd^ grade (convenience sample) - Serbia  *Sampling frame:* Schools in Belgrade region of Serbia  *Sample population*: 11 schools (randomly selected)  *3205 students in 2007 (1660/1545 completed before/after survey)  *2155 students in 2008 (1138/1017 completed before/after survey)  *Demographic characteristics:*  Age (years): generally 15-17  Sex: Not stated (collected in tables)  Skin type: 48% skin that moderately burns and moderately tans, 44% skin rarely burns and easily tans | *Theoretical framework:* None specified  **Intervention “Sunbathing – yes or no?”**  *Format:* Lecture and workshop  *Content:*  *Lecture covered; solar radiation  – wavelengths, damage of the ozone layer and its health-related effects, beneficial effects of sunbathing, adverse effects of UV exposure from sun and/or sunbeds, proper behaviour at the exposure, UVI, sunscreens for beach and daily skin care products, sunbeds – legislation, WHO recommendations, artificial skin tanning products, and post-exposure skin care and products.  *Workshop - students were given several everyday situations and asked to apply what they learnt from the lecture  *Duration:* 90 minutes  *Delivery:* Not stated  **No control** | Six months (2 data collection periods) |
| Pettigrew^27^  2019  Pre/post (pilot study) | *Target population:* High school students (12-18 years) in Perth, Western Australia  *Sample population*:  **Schools** (convenience sample)  *1 IG  *1 CG  **Students**  *IG - 1331  *CG - 1148  *Completing pre/post intervention survey:*  year 7 students  *IG - 221 (77 completed survey)  *CG - 220 (80 completed survey)  *Wearing polysulfone badges – 40 students*  *IG 20  *CG 20  *Observational data – all students potentially*  *Demographic characteristics (those completing surveys):*  Age (years): Year 7 (12-13 years)  Sex: No information  Ethnicity/skin colour: Not provided | *Theoretical framework:* none specified  **Intervention – individually directed plus environmental**  *Format:* Presentation was in assembly, Environmental UVR monitor  *Content:*  *Presentation: purpose of UVI, threshold of 3 for when sun protection is indicated accompanied by graphical content and actionable messages  *Environmental: UVR monitor with attached sign featuring a graduated call to action  *Duration:* Presentation 15 minutes long  *Delivery:* Not stated likely research team  **Control**  Wait listed intervention | 3 weeks |
| Ramstack^47^  Year not provided  Pre/post (uncontrolled) | *Target population:* Delivered to 4-8^th^ grades in Southern Arizona (Note:. only 8^th^ graders (13-14 years) reported in paper)  *Sample population*:  **School**  Assume one school (convenience sample)  **Students**  No numbers provided  *Demographic characteristics:*  Age (years): 8^th^ grade (13-14 years)  Sex: Not provided (for 8^th^ graders)  Ethnicity/skin colour: Not provided (for 8^th^ graders) | **Intervention group**  *Format:* didactic. (workbooks) – interactive and activity based  *Content:* teacher could choose activities from within each unit: The sun, The skin, The sun friend or foe?, Cancer and skin cancer, Prevention of sun damage  *Duration:* 6 units – no details on how frequently delivered or length  *Delivery:* Delivered by classroom teacher (training provided)  **No control** | Follow-up period not provided |
| Swindler^48^  Year not provided  Pre/post (uncontrolled) | *Target population:* High school students in Ohio, USA  *Sample population*:  **Schools**  One high school (convenience sample)  **Students**  **589* students (517 completed follow-up)  *Demographic characteristics:*  Age (years): 13-14 26%, 15-16 62%, >=17 - 12%, Mean 15-16  Sex: 53% Female, 47% Male  Skin type: 9% Type I, 47% Type II-III 36% Type IV, 8% Type V-VI | *Theoretical framework:* Non mentioned  **Intervention group – individually directed**  *Format:* Powerpoint lecture  *Content:* Educational – risks of sun exposure and its contribution to premature aging and skin cancers and effective sun protection including sun avoidance during peak hours, sunscreen knowledge, and sun protective effect of clothing.  *Duration:* 45 minutes  *Delivery:* Forth year medical student  **No control** | 4 months |
| White^49^  2007-2008  Cluster (class) RCT (pilot study) | *Target population:* High school students (13-16 years) in Queensland, Australia  *Sample population*:  **School**  2 secondary schools (1 public, 1 private – convenience sample)  **Class** randomised  Not stated but would assume 1 class in each group  **Students**  *IG – 36 students (loss to FU – 9) CG – 46 students (loss to FU – 17)  *Demographic characteristics:*  Age: Mean age 14.53 +/- 0.69 years  Sex: 59% female  Skin colour/ethnicity: 64% fair skinned | *Theoretical framework:* Theory of Planned behaviour  **Intervention – individually directed**  *Format:* Educational session  *Content*: Belief based intervention (behavioural (advantages and disadvantages), normative (normative beliefs) and control (barrier and motivator) sun safe behaviours.  *Duration*: 1 hour per week for 3 weeks  *Delivery:* Facilitated by Cancer Council Queensland staff  **Control**  No intervention received | 7 months |
